# Draft Genome Sequence of the Mercury-Resistant Bacterium *Acinetobacter idrijaensis* Strain MII, Isolated from a Mine-Impacted Area, Idrija, Slovenia

**DOI:** 10.1128/genomeA.01177-14

**Published:** 2014-11-13

**Authors:** Juan Campos-Guillén, Juan Caballero Pérez, Julio Alfonso Cruz Medina, Carlos Molina Vera, Luz María Salas Rosas, Citlalli Limpens Gutiérrez, Isaac García Salinas, Miriam Rebeca Hernández Ramírez, Gerardo Soto Alonso, Andrés Cruz Hernández, Carlos Saldaña Gutiérrez, Sergio Romero Gómez, Xóchitl Pastrana Martínez, Erika Álvarez Hidalgo, Mateja Gosar, Tatjana Dizdarevič

**Affiliations:** a Facultad de Química, Universidad Autónoma de Querétaro, Cerro de las Campanas s/n, Querétaro, Qro., México; b Facultad de Ingeniería, Universidad Autónoma de Querétaro, Cerro de las Campanas s/n, Querétaro, Qro., México; c Facultad de Ciencias Naturales, Universidad Autónoma de Querétaro, Avenida de las Ciencias s/n, Querétaro, Qro., México; d Geological Survey of Slovenia, Ljubljana, Slovenia; e Idrija Mercury Mine, Idrija, Slovenia

## Abstract

We report here the first draft assembly for the genome of *Acinetobacter idrijaensis* strain MII, isolated from the Idrija mercury mine area (Slovenia). This strain shows a strikingly high tolerance to mercury, and the genome sequence shows genes involved in the mechanisms for heavy metal tolerance pathways and multidrug efflux pumps.

## GENOME ANNOUNCEMENT

Since the late 15th century, the Idrija mercury mine has been one of the largest mercury mines in the world, second only to the mine at Almadén, Spain. Idrija is situated in a narrow valley, 60 km west of the capital city Ljubljana, Slovenia (1). The concentrations of total mercury (THg) in soil samples taken in the 160-km^2^ area influenced by the Idrija mercury mine varied between 3.3 and 973 mg/kg; the maximum permissible concentration (10 mg/kg) set by Slovenian legislation is 19 km^2^ (2). Microbiology studies revealed the presence of mercury-resistant bacteria. One such species is *Acinetobacter idrijaensis*, isolated from soil sampled in the Idrija mercury mine area. *Acinetobacter idrijaensis* strain MII was selected based on its ability to grow in the presence of higher concentrations of HgCl_2_ (200 mg/L) in nutritive medium and for its clear ability to catalyze the volatilization of Hg using the nonradioactive X-ray method (3).

The genome sequence of *Acinetobacter idrijaensis* was obtained using a whole-genome shotgun library strategy using the Illumina HiSeq 2000 sequencing platform at the Macrogen Korea Institute (Seoul, Rep. of Korea). Sequencing was performed with the paired-end strategy to produce 60.4 million reads of filtered sequences; 997 contigs (>100 bp) were assembled with SPAdes version 3.0 (4). The genome of *Acinetobacter idrijaensis* is ~3.95 Mbp in length. The G+C content of the genomic DNA is 36.75%. Genome annotation was done using Glimmer3 (5). A total of 2,367 coding sequences (CDSs) and 62 structural RNAs (58 tRNAs and 4 rRNAs) were predicted using the tRNAscan-SE (6) and RNAmmer version 1.2 servers (7).

Based on rRNA analysis, strain MII is most closely related to *Acinetobacter iwoffi* (99%). Using the genome sequence and predicted gene sets, we investigate the possible mechanism for mercury resistance. The genes (*merB*, *merA*, *merP*, *merT*, and *merR*) involved in Hg reduction and resistance were identified in the genome. Multidrug efflux pump systems were identified (MexB-MexD). Also, the *sdeY* gene was identified, as a multidrug efflux pump, which belongs to the resistance nodulation cell-division (RND) family. The TtgDEF pump was also identified as an RND member able to expel toluene (8). The *czcA* and *czcD* gene complexes, which mediate resistance to Co^2+^, Zn^2+^, and Cd^2+^ by cation efflux, were identified. ABC transporters involved in the uptake of iron, siderophores, heme, sugar, and vitamin B_12_ were identified. Type IV secretion and conjugative transfer systems were also identified. The plasmid stabilization system protein RelE/ParE family was detected. The availability of *Acinetobacter idrijaensis* strain MII genome allows further analysis to gain insight into its pathogenic and metal tolerance, as well as comparisons between *Acinetobacter* genomes.

### Nucleotide sequence accession number.

This whole-genome shotgun project has been deposited in GenBank under the accession number JQCU00000000.
